# Cancer therapy in mice using a pure population of CD8^+^ T cell specific to the AH1 tumor rejection antigen

**DOI:** 10.1007/s00262-021-02912-9

**Published:** 2021-04-01

**Authors:** Marco Stringhini, Ilaria Spadafora, Marco Catalano, Jacqueline Mock, Philipp Probst, Roman Spörri, Dario Neri

**Affiliations:** 1 Department of Chemistry and Applied Biosciences, Swiss Federal Institute of Technology (ETH Zürich), Vladimir-Prelog-Weg 4, 8093 Zurich, Switzerland; 2 Department of Biology, Swiss Federal Institute of Technology (ETH Zürich), Vladimir-Prelog-Weg 4, 8093 Zurich, Switzerland

**Keywords:** Immunotherapy, Adoptive cell therapy, T cells, Immunocytokines, Retroviral antigens

## Abstract

There is a growing interest in the use of patient-derived T cells for the treatment of various types of malignancies. The expansion of a polyclonal and polyspecific population of tumor-reactive T cells, with a subsequent infusion into the same donor patient, has been implemented, sometimes with positive results. It is not known, however, whether a set of T cells with a single antigen specificity may be sufficient for an effective therapy. To gain more insights in this matter, we used naturally occurring T cells recognizing a retroviral peptide (AH1), which is endogenous in many tumor cell lines of BALB/c origin and which serves as potent tumor rejection antigen. We were able to isolate and expand this rare population of T cells to numbers suitable for therapy experiments in mice (i.e., up to 30 × 10^6^ cells/mouse). After the expansion process, T cells efficiently killed antigen-positive tumor cells in vitro and demonstrated tumor growth inhibition in two syngeneic murine models of cancer. However, AH1-specific T cells failed to induce complete regressions of established tumors. The incomplete activity was associated with a failure of injected T cells to survive in vivo, as only a very limited amount of T cells was found in tumor or secondary lymphoid organs 72 h after injection. These data suggest that future therapeutic strategies based on autologous T cells may require the potentiation of tumor-homing and survival properties of cancer-specific T cells.

**Supplementary Information:**

The online version contains supplementary material available at 10.1007/s00262-021-02912-9.

## Introduction

The recent success of immunotherapy in the oncology field has highlighted the important role the immune system plays in controlling tumor growth. On the one hand, therapeutic proteins like recombinant IL2 or immune checkpoint inhibitors may activate tumor-specific CD8^+^ cytotoxic T lymphocytes, thus facilitating the killing of malignant cells in vivo. On the other hand, T cells themselves can be considered as therapeutics in their own right. Different approaches have been investigated, which make use of patient-derived autologous T cells to treat a variety of malignancies [1]. Collectively named adoptive cell therapies (ACTs), these approaches can be divided in two main groups, depending on whether the autologous T cells are genetically manipulated before infusion. Chimeric-antigen receptor T cells (CAR-T cells) and T cell receptor-transgenic cells (TCR-transgenic cells) are two examples of genetically engineered T cells [2]. In both cases, T cells are forced to express a synthetic receptor, specific for a tumor-associated antigen (TAA). CAR-T cells recognize surface antigens thank to their antibody-like receptors, whereas TCR-transgenic cells are equipped with a native T cell receptor and recognize peptides presented by HLA molecules [2]. The extreme paucity of truly tumor-specific surface antigens has so far limited the use of CAR-T cell therapy to certain hematological malignancies, where the toxicity related to the elimination of TAA-positive healthy cells by CAR-T cells (e.g., elimination of CD19-positive B cells) is manageable [3].

Mutated or aberrantly expressed peptides may represent a broader source of tumor-specific antigen, suitable for targeting with TCR-transgenic cells, but such targets are challenging to identify in practice [4]. The practical implementation of TCR-transgenic cell therapies would be further complicated by the need to identify and clone cognate TCRs specific to the patient’s HLA/peptide complex, which would serve as a basis for highly personalized transduction procedures. As an alternative, the use of naturally occurring T cells isolated from the tumor (tumor-infiltrating lymphocytes, or TILs) or blood of the patient has been proposed and clinically investigated [1, 5–8]. ACT with TILs starts with the resection of the tumor of the patient, from which TILs are cultured, enriched for T cells recognizing the tumor or predicted tumor-specific antigenic peptides, and expanded to high numbers [9]. The expanded T cells are then re-infused in the patient in combination with high-dose IL2, following preparative non-myeloablative lymphodepletion [9]. ACT with TILs has been used to treat metastatic melanoma and other solid malignancies [10–13], with objective responses rates in melanoma around 40–50% and complete responses around 10–20% [14]. Toxicity of this type of treatment is mainly related to the patient preconditioning and the administration of high-dose IL2, which are, however, essential for therapeutic activity [15]. Peripheral blood mononuclear cells (PBMCs) have been investigated as another source of tumor-specific T cells for the therapy of melanoma [16]. Cultures of enriched Melan-A-specific CD8^+^ T cells have been successfully obtained from the blood of patients, and the expanded T cells have been shown to survive and accumulate at the site of disease after re-infusion [5, 6]. Interestingly, early stage clinical trials investigating these cells as a treatment showed promising activity when combined with doses of IL2 substantially lower than the ones needed to sustain TILs, and without prior lymphodepletion [5–8].

It would be desirable to study T cell-based therapies in syngeneic murine models, as systematic investigations may lead to the identification of optimal dosing/scheduling strategies or combination opportunities. Mouse models may also be ideally suited to investigate whether a single antigen specificity may be sufficient for the eradication of tumors in vivo, or whether a polyclonal set of polyspecific cells would be required. Until now, these efforts have been limited to experiments with CAR-T cells, TCR-transgenic cells, or tumor-specific T cells isolated from transgenic mice, mostly due to intrinsic difficulties related to the expansion of murine TILs [17–19]. These approaches, however, may fail to adequately mimic the clinical settings, where T cells need to be extensively cultured in vitro before being infused back in the patient.

AH1 is an antigenic peptide presented on H-2L^d^, which was first identified as the immunodominant antigen of the murine colon carcinoma cell line CT26 [20]. It derives from the endogenous murine leukemia virus envelope glycoprotein 70 (gp70), and it is highly expressed in a multitude of murine tumor cell lines of different histological origin, while being virtually undetectable in murine healthy organs [20, 21]. We previously showed that therapy experiment with two different immunocytokines, able to induce complete tumor rejections in BALB/c mice, resulted in an expansion of AH1-reactive CD8^+^ lymphocytes, which protected the host from subsequent challenges with diverse AH1-expressing tumors [21, 22]. Moreover, AH1 showed a therapeutic effect when used as a cancer peptide vaccine [23]. Most BALB/c-derived tumors present AH1 on H-2L^d^, but one cell line (F1F) has previously been characterized as being essentially AH1-negative, thus serving as negative control for therapy experiments [21].

In this article, we report the use of AH1-specific CD8^+^ T cells for adoptive cell therapy. We developed and optimized a protocol for the isolation and expansion of AH1-specific T cells from tumors and from secondary lymphoid organs. We were able to reach up to 470-fold expansion, with purity consistently close to 100% as judged by FACS with AH1-loaded tetramers. Upon recognition of the antigen, the expanded T cells produced IFNγ and TNFα and specifically killed antigen-positive cells in vitro with high efficacy, sparing antigen-negative tumor cells. Administration of AH1-specific CD8^+^ T cells to mice bearing two different syngeneic tumor models resulted in significant tumor growth retardation; however, cures could not be achieved. Our approach, employing a rare population of naturally occurring tumor-specific T cells isolated from donor mice for ACT in syngeneic tumor models, may serve as a basis for future studies on ACT in a model, which closely replicates the procedures used in the clinic.

## Materials and methods

### Animals and tumor cells lines

All the in vivo experiments were conducted under the project license 04/2018, granted by the Veterinäramt des Kantons Zürich (Zurich, Switzerland). Seven- to eight-week-old female BALB/c mice were purchased from Janvier (France). CT26 colon carcinoma (ATCC CRL-2638), WEHI-164 fibrosarcoma (ATCC CRL-1751) and RENCA renal adenocarcinoma (CRL-2947) were obtained from ATCC, and C51 colon carcinoma and F1F fibrosarcoma were kindly provided by M.P. Colombo (Istituto Nazionale Tumori Milan, Italy). 4T1-luc2 mammary carcinoma (Perkin Elmer, former Caliper Life Sciences) was kindly provided by M. Detmar (ETH Zurich, Zurich, Switzerland). Tumor cells were handled and expanded according to supplier’s protocol. Aliquots of cells in complete growing medium containing 10% DMSO were cryopreserved and stored in liquid nitrogen. Authentication including check of post-freeze viability, growth properties and morphology, test for mycoplasma contamination, isoenzyme assay and sterility test was performed by the cell bank before shipment. Tumor cells were kept in culture for no longer than 2 weeks.

### Immunocytokine production

The recombinant fusion proteins L19-IL12 and F8-IL2 were expressed in stably transfected CHO-S cells, as already described [24, 25]. Fusion proteins were purified by Protein A chromatography and stored in phosphate buffer saline pH 7.4 (PBS) at −80 °C. Characterization of the product included SDS-PAGE under reducing and non-reducing conditions, size exclusion chromatography (S200 10/300 increase column, GE Healthcare) and LC–MS after treatment with PNGase F (NEB), following supplier’s protocol.

### Reversible multimers production

Reversible, AH1-peptide-loaded H-2L^d^ multimers were obtained as described elsewhere [26], with minor adaptations. Briefly, C-terminal Histidine-tagged murine H-2L^d^ and human b2-microglobulin were expressed in *E. coli* and assembled into MHC class I monomers in the presence of AH1 peptide following established protocols. AH1 peptide was obtained from Biomatik. Synthesis of the biotinylated, NTA-containing scaffold (here referred as MVP compound) is described in Supplementary Data. MVP compound was incubated in PBS containing NiSO_4_ (final concentration 10 mM) for 1 h at 4 °C. 0.25 molar equivalent of PE-conjugated Streptavidin (BioLegend) was added. After 1 h incubation at 4 °C, followed by 1 h at RT, AH1-loaded, Histidine-tagged H-2L^d^ monomers, purified by size exclusion chromatography, were added (1:1 molar equivalent with respect to MVP compound) and newly formed multimers were stored at 4 °C (for at least 48 h, before use).

### Antibodies and reagents for flow cytometry

Fluorophore-conjugated antibodies against murine CD8a (53–6.7), CD62L (MEL-14), CD44 (IM7), PD-1 (29F.1A12), CTLA-4 (UC10-4B9), Tim-3 (RMT3-23), Lag-3 (C9B7W), CD25 (PC61), CD11c (N418), CD11b (M1/70), XCR1(ZET), CD80 (16-10A1), IL12 (C15.6), CD137L (TKS-1), CD215 (6B4C88), CCR7 (4B12), CXCR3 (CXCR3-173), as well as fluorophore-conjugated Streptavidin, TruStain FcX mouse anti-CD16/32 antibody (93) and reagents for life/dead discrimination 7-AAD and ZombieRed were all purchased from BioLegend. Anti-TNFα (TN3-19.12) and anti-IFNγ (XMG1.2) were purchased from eBiosciences. Anti-IL2 (JES6-5H4) and anti-CD212 (114) were purchased from BD Biosciences. Anti-iNOS2 (sc-7271) was purchased from Santa Cruz Biotechnology. PE-conjugated, peptide-loaded MHC class I tetramers were produced in-house as already described [21].

### Therapy experiments

Eight- to Nine-week-old female BALB/c mice were subcutaneously injected with 3 × 10^6^ CT26 (or 2.5 × 10^6^ WEHI 164) tumor cells in the right flank. Animals were inspected daily; body weight was monitored, and tumor size was determined using the following formula: tumor volume [mm^3^] = 0.5 × (major diameter) × (minor diameter)^2^. All therapy experiments were initiated when tumors reached a volume of about 100mm^3^. Pre-therapy to boost tumor-specific T cells consisted in a total of three intravenous injections of 15 μg L19-IL12 at 48 h intervals. Mice were sacrified 48–72 h after the last injection to isolate T cells. Prior to ACT experiments, mice were randomized. ACT was performed with in vitro expanded T cells, repeatedly washed and resuspended in PBS before administration, without any preconditioning of the animals. In combination experiments only, mice received F8-IL2, as described in corresponding figures.

### T cells isolation

Tumors, spleens and tumor-draining lymph nodes (right inguinal, right axillary) of L19-IL12-treated mice were harvested. Single cells suspensions of each tissue were obtained as already described [27]. In some cases, splenocytes and lymphocytes from TDLNs were pooled and processed together. Splenocytes were treated with MojoSort Mouse CD8 Isolation kit (BioLegend), following supplier’s protocol, and enriched CD8^+^ T cells were incubated for 15′ at 4 °C with 1.4 μM Avidin to quench residual free Biotin in the sample, which could bind to Streptavidin in reversible multimers. Cells suspensions were stained with fluorophore-conjugated anti-CD8a and PE-conjugated reversible multimers for 30′ at 4 °C; 7-AAD was used following supplier’s protocol to exclude dead cells. Multimer-positive CD8^+^ T cells were sorted on a FACS Aria II. Cells were collected in tubes or wells containing Complete Medium (CM: RPMI-1640 supplemented with 10% FBS, 1xAntiAnti, 50 μM BME, 25 mM HEPES, 4 mM Ultraglutamine) supplemented with 100 mM Imidazole.

### Preparation of dendritic cells

Dendritic cells were derived from bone marrow cells with established protocols. Briefly, bone marrow was harvested from femurs and tibiae of BALB/c mice, treated with red-blood cells lysis buffer (BioLegend) and plated in R10 medium (RPMI-1640 supplemented with 10% heat-inactivated FBS, 1xAntiAnti, 12.5 mM HEPES, 4 mM Ultraglutamine) supplemented with either 20 ng/mL murine GM-CSF (BioLegend) or 200 ng/mL murine Flt3L (eBiosciences). Cells grown in GM-CSF were plated in 100 mm non-adherent petri dishes at a density of 2.5 × 10^5^ cells/mL in 10 mL, whereas cells grown in Flt3L in wells of 6-well-plates at a density of 1.5–2 × 10^6^ cells/mL. After 3 days, 5 mL R10 supplemented with 20 ng/mL murine GM-CSF were added to cultures grown in GM-CSF. Cells grown in GM-CSF were activated overnight at day 6, with 1 μg/mL LPS (Sigma), whereas cells grown in Flt3L were activated overnight at day 9, with 1 μg/mL poly I:C (GE Healthcare). Non-adherent cells were harvested, resuspended in R10 containing 10% DMSO and cryopreserved in liquid nitrogen.

### T cells culture with IL2 and dynabeads

Multimer positive CD8^+^ T cells from tumors and TDLNs, respectively, multimer negative CD8^+^ T cells from TDLN of BALB/c-bearing CT26 tumors were isolated as described above. After washing, cells were resuspended in CM containing 60 IU/mL Proleukin (Novartis) and Dynabeads Mouse T-Activator CD3/CD28 (Thermofisher, 1:1 Dynabeads-to-T cells) and incubated at 37 °C, 5% CO_2_. Plate was incubated tilt for the first 6 h. Half of the medium with fresh Proleukin was replaced every three days. Cells were resuspended and distributed in additional wells when confluent. Pictures were taken on a Zeiss AxioVert 200 M equipped with a AxioCam MRm, using AxioVision software (version 4.8.2) at days 1, 3 and 7. Flow cytometry analysis was performed at day 7.

### Expansion of T cells

Sorted T cells were washed once with CM supplemented with 100 mM Imidazole and once with CM, before being plated in pre-warmed CM supplemented with 100 ng/mL IL15. Cells were plated in wells of either 96- or 48-well-plates, and plates were incubated at 37 °C, 5% CO_2_ for 5 days (tilt for the first 12 h). T cells were activated at day 5 by adding peptide-pulsed DCs (1:5 to 1:1 DCs-to-T cells) suspended in CM supplemented with IL15. DCs were thawed the night before activation, let rest in R10 for 12–14 h and activated with 1 μg/mL LPS or poly I:C for 6 h. During the last hour, DCs were pulsed with 1–5 μM AH1 peptide. Before adding them to T cells, DCs were washed in CM. One-half of the medium was replaced every 3–4 days. T cells were re-activated at day 15, and 10 ng/mL IL7 was added to culture medium from day 15 onwards. One-half of the medium was replaced every 3–4 days, and cells were split in additional wells whenever confluent. T cells were harvested and used for analysis and therapy between days 21 and 24. In some experiments, T cells were expanded in the presence of either 0.5 μM Doramapimod (Cayman Chemical), 7 μM TWS119 (Sigma) or 2.5 μg/mL mDLL1-Fc (R&D Systems).

#### Flow cytometry analysis

For flow cytometry analysis, T cells were stained for 30′ at 4 °C in FACS buffer with fluorophore-conjugated antibodies against CD8a, CD62L, CD44, PD-1, Tim-3, Lag-3, CTLA-4, CD212, CD25, CCR7, CXCR3 and with PE-conjugated, peptide-loaded tetramers. Dead cells were excluded from analysis by 5′ staining in 7-AAD at 4 °C.

Bone marrow-derived, AH1 peptide-pulsed dendritic cells cultured and matured in different conditions were stained using the same protocol, with antibodies against CD11c, CD11b, XCR1, PD-L1, CD137L, CD80 and CD215, after Fc-Receptor blocking with TruStain FcX mouse anti-CD16/32 (following supplier’s protocol). For intracellular staining of DCs, cells were first stained with ZombieRed (1:500 in PBS), for 15′ at RT. After surface staining, cells were treated with the Foxp3/Transcription Factor Staining Buffer Set (eBiosciences), as described by the supplier and stained with antibodies against IL2, IL12 and NOS2. In some cases, dendritic cells were pulsed with a FITC-lated version of the AH1-peptide (produced in-house as described in Supplementary data). Cells were analyzed on a CytoFlex S (Beckman Coulter) and data processed with FlowJo software (version 10).

#### Cytokine release assay

T cells were incubated either alone, with Dynabeads Mouse T-Activator CD3/CD28 (Thermofisher) or with tumor cells (CT26 and F1F) at a 1:1 ratio in CM. After 1 h incubation at 37 °C, Brefeldin A (BioLegend) was added to the samples (final concentration 5 μg/mL). After 4–5 h in incubator, cells were harvested for staining and flow cytometry analysis. Beads were removed with a magnet before staining. Cells were stained in ZombieRed, followed by staining with anti-CD8a. Cells were fixed and permeabilized using the Foxp3/Transcription Factor Staining Buffer Set (eBiosciences), following supplier’s protocol, and subsequently stained with fluorophore-conjugated antibodies against TNFα, and IFNγ, for 30′ at 4 °C. Fluorescence Minus One (FMO) controls were used to define negative populations.

#### Cell cytotoxicity assay

T cells and target tumor cells at different effector-to-target ratios in CM were incubated in a static incubator at 37 °C for 24 h. Cells were spun down, medium was removed completely, and cells were washed once with PBS. Adherent tumor cells were detached using 0.05% Trypsin/EDTA solution (Thermofisher), and CM was added to neutralize trypsin. Cells were pelleted, washed in FACS buffer and stained with fluorophore-conjugated anti-CD8a and 7-AAD. Cells were analyzed on a CytoFlex S (Beckman Coulter) and data processed with FlowJo software (version 10). Living, CD8^+^ T cells were plotted together with living CD8-negative tumor cells.

#### In vivo biodistribution

In vitro expanded T cells were harvested and stained using the CFSE Cell Division tracker kit from BioLegend, following supplier’s protocol. The number of living T cells was determined by Trypan Blue exclusion using a hemocytometer, and 3 × 10^6^ CFSE-labeled T cells were intravenously injected in BALB/c-bearing established subcutaneous CT26 tumors. An aliquot of labeled T cells was stained with ZombieRed and anti-CD8a and analyzed by flow cytometry to determine viability and intensity of CFSE signal at baseline. After 72 h, mice were euthanized and tumors, TDLNs and spleens were harvested and processed to single cell suspensions. Samples were stained with ZombieRed and fluorophore-conjugated antibodies against CD8a and analyzed by flow cytometry.

#### Statistical analysis

Data were analyzed using Prism 7.0 (GraphPad Software, Inc.). Statistical significance in tumor therapies was determined with a regular two-way ANOVA test with Bonferroni post-test correction. All the other statistical analyses were performed using a two-tailed, unpaired Student’s *t* test. *P* < 0.05 was considered statistically significant.

## Results

### AH1-specific CD8^+^ T cells do not expand in standard culture conditions

**Fig. 1:**
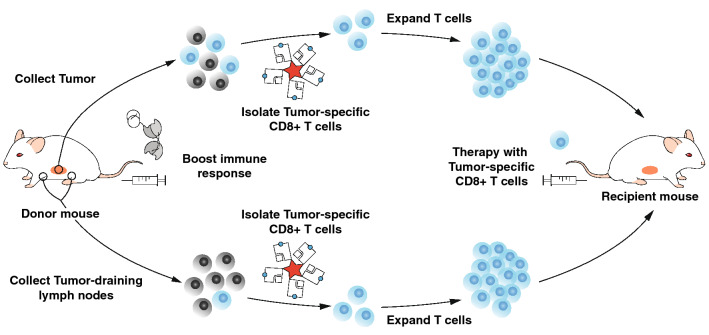
Workflow. Strategy to isolate and expand AH1-specific TILs or T cells from secondary lymphoid organs for ACT

**Fig. 2:**
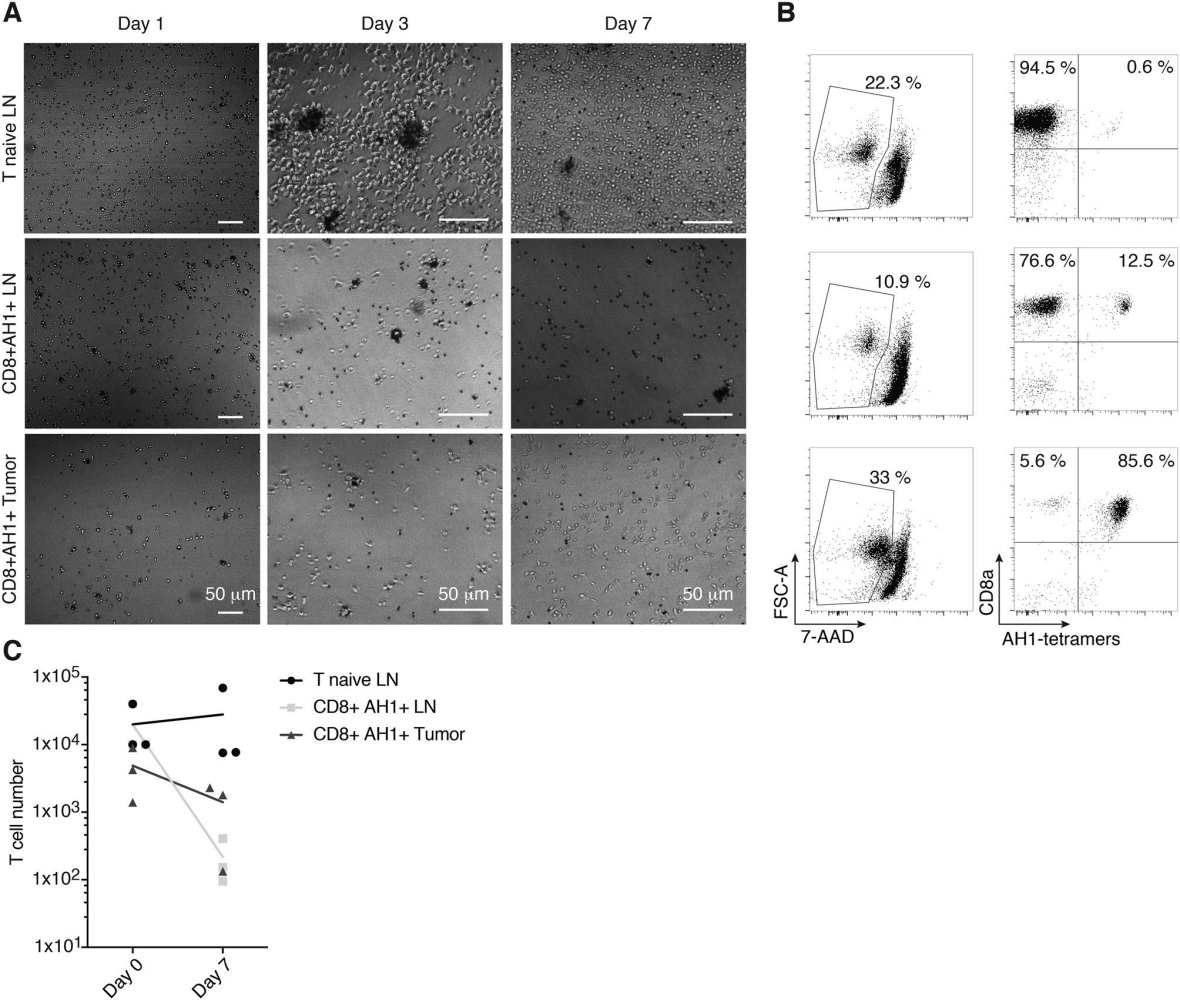
T cell culture with anti-CD3, anti-CD28 and IL2. AH1-specific T cells from tumors (CD8^+^ AH1 + tumor) or TDLNs (CD8^+^ AH1 + LN) were isolated by FACS and grown with activating magnetic beads and IL2 for 7 days. Tetramer-negative T cells isolated similarly from TDLNs (T naïve LN) were used as control. **a** Representative picture of T cells was taken at days 1 (5 × magnification), 3 and 7 (10 × magnification). **b** Representative flow cytometry analysis, performed at day 7. **c** T cells count at baseline (day 0) and after 7 days in culture. T cells were obtained from 3 different mice. Data are representative for 2 independent experiments

In order to be able to perform ACT with a pure population of AH1-specific CD8^+^ T cells, these cells had to be isolated and expanded in vitro (Fig. 1). We used peptide-loaded MHC class I reversible multimers to isolate AH1-specific CD8^+^ T cells from TDLNs and tumors of CT26-bearing BALB/c mice. As control, we isolated AH1-multimer-negative CD8^+^ T cells from TDLNs (T naïve). We used magnetic beads coated with antibodies against CD3 and CD28 to activate the T cells and grew them in complete medium supplemented with IL2. After three days in culture, T naïve had formed big clumps around beads and were increased in size and number (Fig. 2a). Some small clumps of cells and beads were also present in wells containing AH1-specific T cells, but these cells were smaller and did not seem to have increased in number. Microscopic analysis was repeated at day 7. T naïve had returned to normal size, but maintained an elongated shape typical of proliferating cells and further increased in number. AH1-specific cells did not seem to have expanded (Fig. 2a). Cells were collected, counted and analyzed by flow cytometry. Viability was low in all samples (Fig. 2b), but while the total number of T naïve was comparable or higher than at culture initiation, AH1-specific T cells from both TDLNs and tumors had significantly decreased (Fig. 2c). Interestingly, the percentage of AH1-tetramer-positive T cells was also decreased with respect to baseline in AH1-specific T cells samples derived from TDLNs, but not from tumors. This effect was more marked in less diluted samples, where T cells and beads were in closer contact and thus more likely to interact. Collectively, our findings evidenced a clear need for better experimental procedures, which would allow the selective expansion of antigen-specific CD8^+^ T cells.

### AH1-specific CD8^+^ T cells can be expanded by repetitive stimulation with AH1 peptide-pulsed mature dendritic cells

**Fig. 3:**
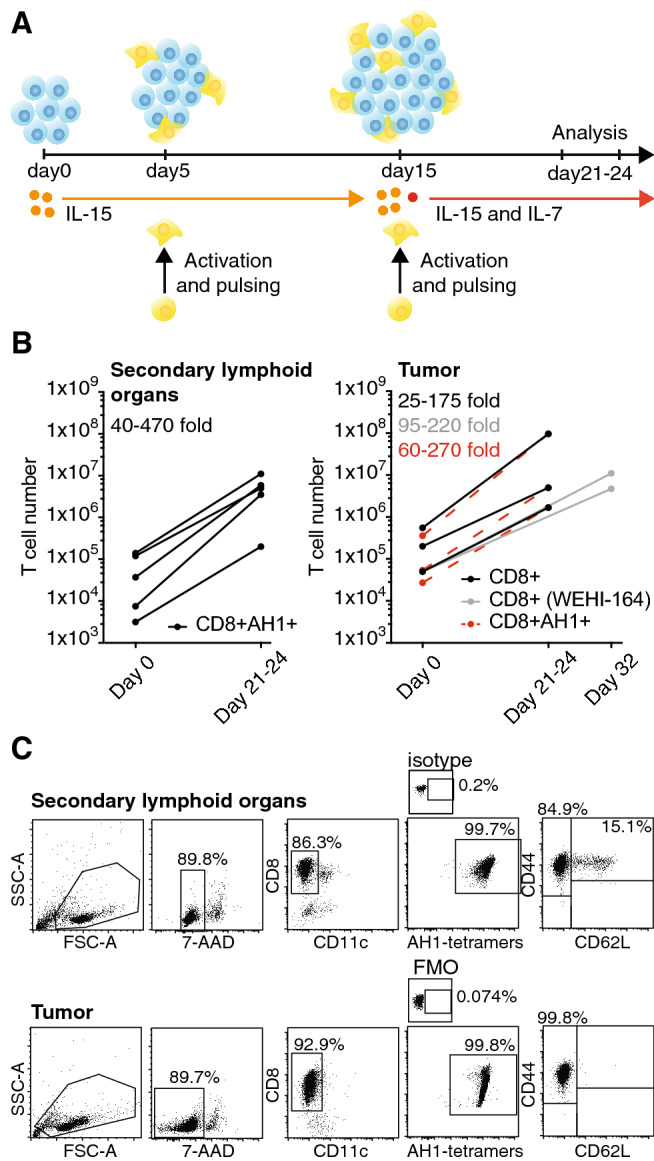
Optimized protocol for the expansion of AH1-specific T cells. **a** Schematic representation of the protocol used to expand AH1-specific T cells. **b** T cell count at baseline (day 0) and after 3–4 weeks of in vitro expansion. Each line represents one independent experiment, AH1+ CD8^+^ T cells were isolated by FACS from secondary lymphoid organs (left) and CD8^+^ TILs from CT26 or WEHI tumors (right). Scattered lines represent baseline counts of AH1-positive cells in CD8^+^ TILs samples. **c** Representative flow cytometry analysis of T cells after expansion. PE-conjugated tetramers loaded with an irrelevant peptide (p29) or Fluorescence Minus One (FMO) controls were used to set the AH1-tetramers gate

In vitro expansion leads to a gradual maturation of T cells toward a terminally differentiated phenotype. Naïve-like or minimally expanded T cells have been shown to engraft more efficiently in vivo, leading to an increased anti-tumor effect [28]. Many studies have reported that culturing naïve T cells in IL15 and IL7 results in less exhausted or more naïve-like T cells, with respect to expansion in IL2 [28]. With preliminary experiments, in which we cultured CD90.2 + BALB/c splenocytes with activating beads and cytokines, we could observe that CD8^+^ T cell yields and the proportion of CD8^+^ T cells expressing CD62L increased upon use of IL7 and IL15, rather than IL2 (Supp. Figure 6). Also, culture in IL2-containing medium required re-activation every 5–6 days, whereas culture in IL15 and IL7 did not. Teague et al*.* had previously shown that tumor-specific tolerant T cells, which failed to proliferate in vitro, could be rescued by incubation in medium containing high-dose IL15 (or, less efficiently, IL2) [29]. After isolation of AH1-specific T cells from secondary lymphoid organs and tumor, we incubated cell samples in complete medium supplemented with high-dose IL15 and let them rest for five days. At day five, we stimulated the T cells using peptide-pulsed, mature BMDCs expressing various co-stimulatory molecules (Supp. Figure 7) and we repeated the stimulation after 10 days. After the second stimulation, we started adding IL7 in the medium. Following a slow but constant expansion for the first two weeks, T cells started to expand exponentially about three to four days after the second stimulation. Exponential growth usually lasted 4–5 days, after which proliferation rate slowed down. Figure 3a graphically depicts the protocol used to expand AH1-specific T cells. Using this protocol, we were able to reliably expand T cells up to 470-fold in 3 weeks (Fig. 3b). Remarkably, almost 100% of the CD8^+^ T cells were able to bind AH1-loaded MHC I tetramers after the expansion (Fig. 3c).

Activation of unselected CD8^+^ TILs with peptide-pulsed DCs, but not with DCs and anti-CD3, led to a preferential expansion of AH1-specific T cells (Supp. Figure 8). This effect was not only observed with CD8^+^ TILs from CT26 tumors, where AH1-specific cells represented about 50–60% of the total T cells, but also with CD8^+^ TILs from WEHI-164 tumors, where the initial percentage of AH1-specific cells was consistently lower than 10%. CD8^+^ TILs isolated from WEHI-164 tumors required a third stimulation before starting to expand exponentially (Fig. 3b). Expanded T cells had an effector phenotype (CD62L-/CD44high) and expressed the IL2Rα subunit CD25. Diverse markers of exhaustion were found to be heterogeneously expressed among expanded T cells (Fig. 3c and Supp. Figure 8).

### In vitro expanded AH1-specific CD8^+^ T cells are functional and selectively recognize tumor cells expressing gp70

**Fig. 4:**
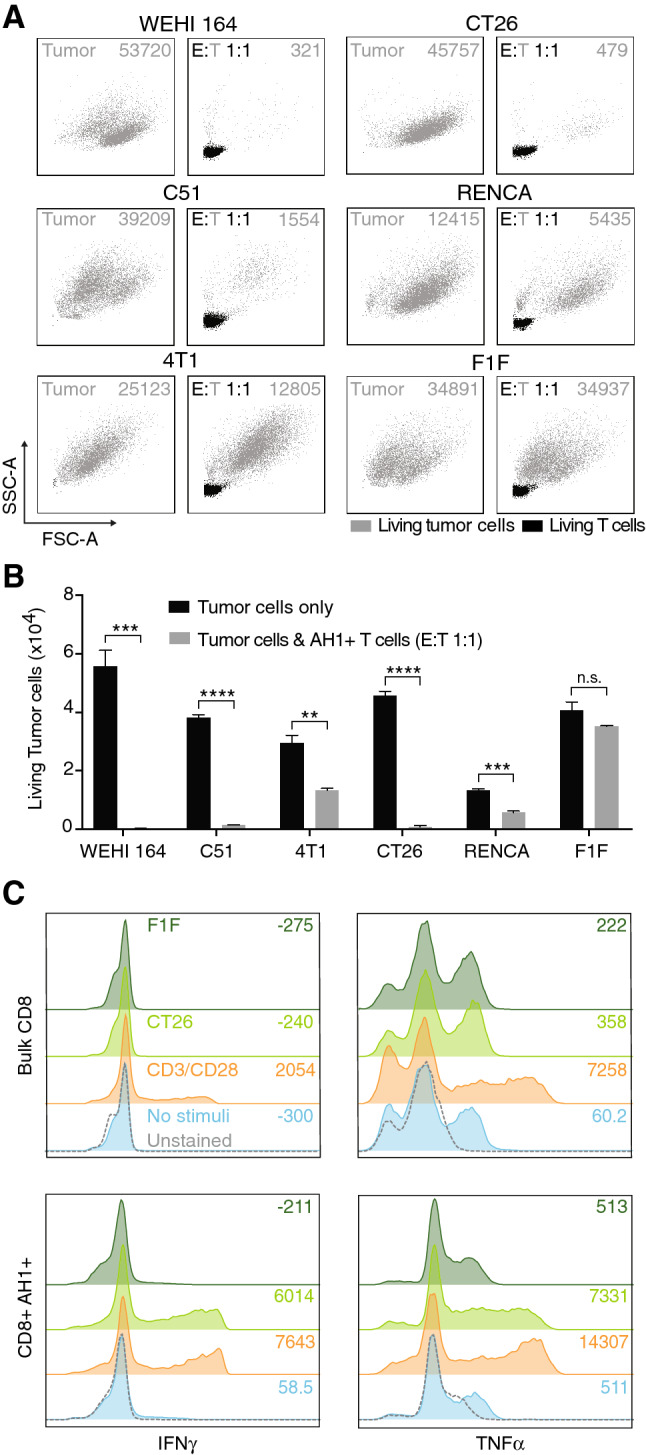
In vitro activity of AH1-specific T cells. **a** Different tumor cell lines were incubated for 24 h, either alone (left boxes) or with AH1-specific T cells at a 1:1 ratio (right boxes) and analyzed by flow cytometry. Representative graphs showing living tumor cells (gray) and living CD8^+^ T cells (black). Numbers on the top right corner of the boxes represent count of living tumor cells. **b** Total count of living tumor cells with or without addition of T cells. Column represents means + SEM, *n* = 3 per experimental group, ns = non-significant, *p < 0.05, **p < 0.01, *** *p* =  < 0.001, **** *p* < 0.0001 (unpaired *t* test). **c** Flow cytometry analysis of expanded CD8^+^ AH1^−^ or CD8^+^ AH1^+^ T cells stimulated with antiCD3/antiCD28-coated magnetic beads, F1F or CT26 tumor cells (all at 1:1 ratio). Numbers on the right sides of the boxes represent mean fluorescence intensities. Data representative for at least five independent experiments

In order to check whether expanded T cells were functional, we tested their biocidal capacity in an in vitro cytotoxicity assay. We incubated expanded T cells with different tumor cell lines, which were shown to express the gp70 protein (from where the AH1 peptide is derived) or with F1F tumor cells, which do not express gp70 [21, 30]. Already at a 1:1 ratio, AH1-specific T cells were able to selectively eliminate gp70-expressing tumor cells, with variable efficiency (Fig. 4a, b). CT26, WEHI-164 and C51 were the most sensitive cell lines and were almost completely eliminated by T cells after 24 h. To test the ability of expanded T cells to secrete effector cytokines, we isolated AH1-specific CD8^+^ or bulk CD8^+^ T cells from secondary lymphoid organs, we expanded them using suitable protocols and we incubated them with CT26 cells, F1F cells or activating anti-CD3/anti-CD28 beads. Flow cytometry analysis showed that bulk CD8^+^ T cells (containing negligible levels of AH1-specific T cells, Supp. Figure 9) were able to produce IFNγ and TNFα upon incubation with beads, but not with tumor cells. AH1-specific T cells, instead, produced IFNγ and TNFα both upon stimulation with beads and CT26, but not with F1F tumor cells (Fig. 4c).

### In vivo administration of AH1-specific T cells delays tumor progression in two murine models of cancer

**Fig. 5:**
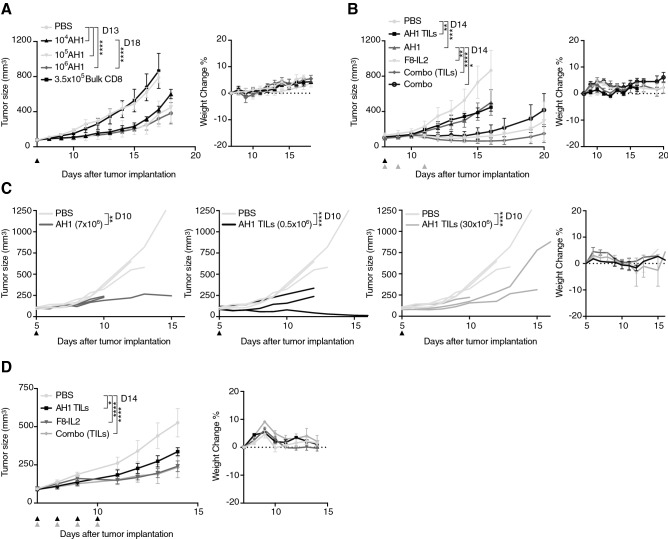
Tumor therapy with AH1-specific T cells. **a** Tumor growth over time with respective weight change, in BALB/c-bearing CT26 tumors and treated with one intravenous injection (*i.v.*) of AH1-specific T cells or CD8^+^ AH1^−^ T cells (bulk CD8) expanded from secondary lymphoid organs. Data represent means ± SEM, *n* = 5 per experimental group. **b** Same as in **a**, but mice received either saline (PBS), 5 × 10^5^ AH1-specific T cells expanded either from secondary lymphoid organs (AH1) or from tumors (AH1 TILs), 3 × 45ug F8-IL2, or combinations of T cells and F8-IL2, as indicated by arrows. Data represent means ± SEM, *n* = 5 per experimental group. **c** Therapy experiment in BALB/c-bearing WEHI-164 tumors and receiving either saline, or the indicated doses of AH1-specific T cells from TDLNs (AH1) or tumors (AH1 TILs). Data represents single mice, *n* = 3 per experimental group. Some mice had to be euthanized before the end of the therapy because of tumor ulceration, in accordance to the license 04/2018. **d** Therapy in CT26-bearing BALB/c and receiving 4 × *i.v.* injections of either saline, 5 × 10^5^ AH1-specific TILs, 20ug F8-IL2 or 5 × 10^5^ AH1-specific TILs pre-mixed with 20ug F8-IL2 before administration. Data represent means ± SEM, *n* = 5 per experimental group. Statistical analyses were performed on the day indicated in the legends, ns = non-significant, * *p* < 0.05, ** *p* < 0.01, *** *p* =  < 0.001, **** *p* < 0.0001 (2-way ANOVA with Bonferroni correction)

Using our protocol based on pulsed dendritic cells and a cocktail of IL7 plus IL15, we were able to successfully expand functional AH1-specific CD8^+^ T cells. In order to investigate their therapeutic potential in vivo, we performed a first therapy experiment in BALB/c mice bearing subcutaneous CT26 tumors. We administered increasing doses of AH1-specific T cells by intravenous injections. As negative control, two additional groups of mice were injected with saline, resp. bulk CD8^+^ T cells containing negligible levels of AH1-specific T cells (Supp. Figure 9). Administration of AH1-specific T cells at all tested doses leads to a significant tumor growth retardation compared to both the saline and the bulk CD8^+^ group (Fig. 5a). Therapy with 1 × 10^6^ T cells performed substantially better than therapy with 1 × 10^4^ T cells, but not than therapy with 1 × 10^5^ T cells. By contrast, therapy with 3.5 × 10^5^ bulk CD8^+^ T cells did not show any anti-tumor effect compared to saline. All treatments were well tolerated, with no mice experiencing weight loss. In order to boost the activity of AH1-specific T cells and to assess whether their origin could influence therapeutic potential, we performed a second experiment in the same model, where we combined AH1-specific CD8^+^ T cells from secondary lymphoid organs or tumors with human IL2. More precisely, we used a targeted version of IL2 genetically fused to the antibody F8, specific for the tumor-associated antigen Extra Domain A of fibronectin [25]. We used this version of IL2 with the goal of maximizing tumor-over-healthy organs exposure. AH1-specific T cells mediated tumor growth retardation regardless of their tissue of origin, and the effect was in line with what observed in the first therapy experiment (Fig. 5b). Administration of the T cells together with F8-IL2 leads to a significantly improved anti-tumor effect, which was, however, mainly mediated by F8-IL2. We could not observe any significant difference between combination treatments and treatment with F8-IL2 alone. In a third therapy experiment, we investigated the effect of AH1-specific T cells in a second model, the WEHI-164 fibrosarcoma. With the goal of investigating whether an increase in the number of injected T cells would improve anti-tumor activity, we devoted considerable efforts to obtaining higher yields of AH1-specific T cells. We were able to administer up to 30 × 10^6^ T cells to three mice, but surprisingly we did not observe any further therapeutic improvement compared to previous experiments (Fig. 5c). We hypothesize that absence of therapy improvement upon administration of a high number of T cells may be due to an insufficient survival of those cells in vivo. In fact, AH1-specific cells had been extensively expanded in vitro, possibly impairing their subsequent proliferative fitness. Moreover, availability of supportive cytokines, growth factors and nutrients for the newly infused T cells may be limited due to competition with lymphocytes already present in the tumor and tumor cells themselves, allowing only a limited amount of administered T cells to survive. It has already been demonstrated that lymphodepletion prior to ACT substantially improves engraftment of infused T cells and, consequently, their efficacy [31, 32]. As an attempt to find an alternative strategy to improve anti-tumor effect without employing lymphodepletion, we performed an additional therapy in CT26, using a modified administration schedule. Instead of administering T cells as a single bolus injection, we injected low doses of T cells for four consecutive days, alone or in combination with F8-IL2. Unfortunately, we did not observe any significative improvement compared to the previous therapies (Fig. 5d).

### Administered T cells fail to persist in tumor or secondary lymphoid organs

**Fig. 6:**
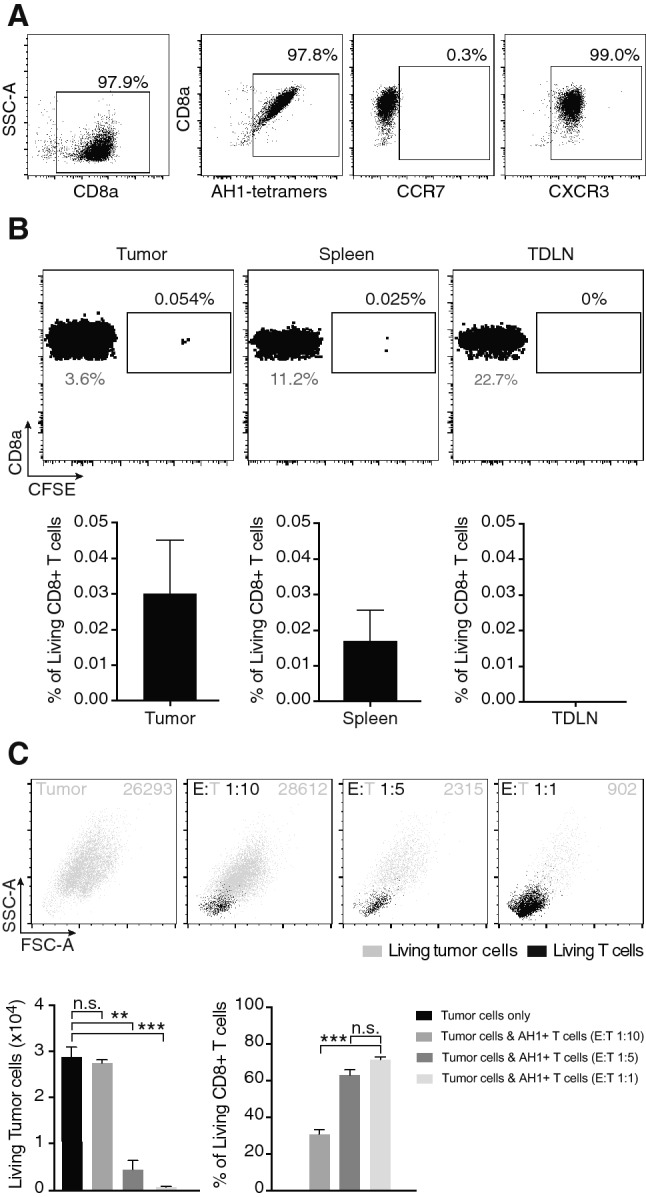
In vivo biodistribution and in vitro survival of AH1-specific T cells. **a** Expression of chemokine receptors CCR7 and CXCR3 on expanded AH1-specific TILs, analyzed by flow cytometry. **b** 3 × 10^6^ CFSE-labeled AH1 + TILs were *i.v.* injected in BALB/c-bearing established CT26 tumors. Mice were sacrified after 72 h, and presence of CFSE+ CD8^+^ T cells was analyzed in tumors, TDLNs and spleens. Representative dot plots showing fractions of CFSE+ cells over total CD8^+^ cells in each tissue, with respective bar plots, representing means ± SEM (*n* = 3 mice). Numbers in gray represent the percentage of CD8^+^ cells over total living cells for each tissue. **c** Representative flow cytometry analysis from a cell-cytotoxicity assay featuring CT26 tumor cells and AH1-specific TILs at indicated effector-to-target ratios, with respective bar plots. Bar plots represent total number of living tumor cells (left) and percentage of living CD8^+^ T cells on total CD8^+^ T cells (right), for each experimental group (means ± SEM, *n* = 3 per experimental group). ns = non-significant, *p < 0.05, **p < 0.01, *** p =  < 0.001, **** *p* < 0.0001 (unpaired *t* test)

Numerous studies have reported the ability of in vitro expanded T cells to accumulate in the tumor mass [33, 34]. Mechanistically, T cells ability to migrate into the tumor after intravenous administration depends on expression of various adhesion molecules and chemokine receptors. CXCR3 is expressed on activated TILs found in various cancer types, and it is thought to play an important role in T cell migration to inflamed tissues [35]. Flow cytometry analysis revealed that expanded AH1-specific CD8^+^ T cells homogeneously expressed CXCR3, but lacked expression of the secondary lymphoid-homing chemokine receptor CCR7 (Fig. 6a). We performed an in vivo biodistribution study by injecting CFSE-labeled AH1-specific T cells into BALB/c-bearing subcutaneous CT26 tumors, in order to evaluate whether T cells were able to engraft and persist in vivo. Analysis of single cell suspensions from tumors, TDLNs and spleens 72 h after administration of 3 × 10^6^ T cells revealed the presence of extremely few CFSE-positive CD8^+^ T cells in tumors and spleens only, with CFSE-positive T cells reaching less than 0.1% of the total CD8^+^ T cells in those tissues (Fig. 6b). With so few adoptively transferred T cells present in the tumor (i.e., the site in which they would be expected to exert their activity) and in secondary lymphoid organs (where they could potentially proliferate and replenish the TIL-pool), it may be difficult to achieve a sufficiently potent and sustained anti-tumor effect. In support of this hypothesis, in vitro cytotoxicity experiments with T cells-to-tumor cells ratios lower than 1:2 (which may better mimic the in vivo situation) revealed that effector-to-target ratios as low as 1:10 not only did not have any effect on tumor cells growth, but negatively affected T cells viability (Fig. 6c).

### Expansion of TILs in the presence of Notch-ligand or inhibitors of GSK-3b and p38 does not results in T cells with favorable phenotype

**Fig. 7:**
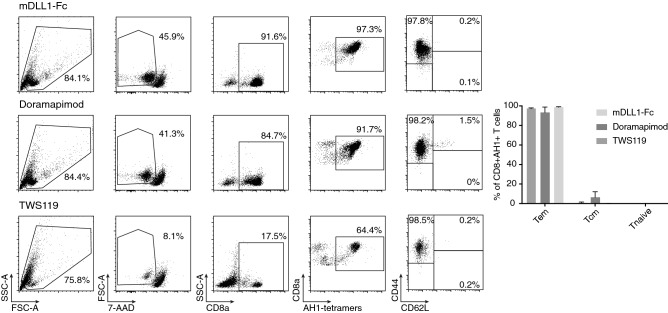
TILs expansion in the presence of additives. Representative phenotypic analysis of TILs expanded in the presence of recombinant Notch-Ligand (mDLL1-Fc), p38 inhibitor Doramapimod or GSK-3b inhibitor TWS119, with respective bar plots. (Columns represent means ± SEM, *n* = 3 per experimental group.) T_cm_ = central memory T cells (CD44high/CD62^+^), T_em_ = effector memory T cells (CD44high/CD62^−^) T_naive_ = naïve T cells (CD44low/CD62^+^)

Culture protocols employing GSK-3b inhibitor TWS119 or, more recently, p38 inhibitor Doramapimod have been effectively employed to expand naïve tumor-specific T cells, while limiting their maturation [36, 37]. The so-obtained T cells maintained naïve- or stem cell-like characteristics and were shown to perform better in ACT experiments. Another study reported that Notch-signaling during culture of activated T cells could increase the proportion of CD62L + naïve or central memory T cells [38]. In order to test whether those agents could also reprogram antigen-experienced T cells to become more naïve-like and thus more fit for survival in vivo, we cultured TILs in the presence of each of these three agents. T cells cultured in the presence of Doramapimod and recombinant Notch-ligand efficiently expanded, but phenotype analysis based on expression of CD44 and CD62L revealed that none of the additive was able to reliably expand T cells with a central memory or naïve-like phenotype (Fig. 7). Prolonged exposure to TWS119 was toxic for T cells.

## Discussion

In the present study, we were able to develop a protocol for the reliable expansion of AH1-specific T cells isolated from natural infiltrates of tumors and secondary lymphoid organs. The expanded T cells maintained their specificity, recognized and killed antigen-positive tumor cells with high efficiency in vitro and delayed tumor progression when used as ACT in two syngeneic murine models of cancer. When used alone, AH1-specific T cells failed to induce regression of established tumors in immunocompetent mice. Combination with F8-IL2, a tumor-targeted version of human IL2, did result in substantially improved therapeutic effect, which was, however, not superior to F8-IL2 monotherapy, suggesting that IL2 preferentially acted on the preexisting immune infiltrate, rather than on injected T cells.

Since the number of infused T cells positively correlates with therapeutic activity in patients, it may be argued that curative results were not obtained because an insufficient number of AH1-specific T cells were used. We investigated doses ranging from 1 × 10^4^ to 30 × 10^6^ AH1-specific T cells. We cannot exclude that a higher number of T cells may be sufficient to obtain complete rejections. Similar experiments in a different model showed how established tumors could be completely eradicated by increasing the doses of administered tumor-specific T cells [17, 39]. Such studies, however, were performed using T cells from transgenic mice, which gives the authors access to very high numbers of naïve tumor-specific T cells, an ideal but unrealistic scenario. It is common knowledge that expanding murine TILs (and, as we showed, antigen-experienced, tumor-specific T cells from lymphoid organs) is not trivial and the yields of these *bona fide* impaired T cells one can obtain may be limited. Although expanding human TILs may be easier than expanding murine TILs, in both species, naturally occurring tumor-specific T cells are rare. Extensive in vitro expansion with current protocols, needed to obtain high cell numbers, inevitably drives the T cells to a gradual loss of survival and proliferation potential [40]. Thus, also the number of human TILs one can obtain for therapy may be limited. This, together with the fact that we did not observe any improvement by injecting up to 30 × 10^6^, compared to 0.5 × 10^6^ AH1-specific T cells, may suggest that focusing on alternative strategies, rather than on trying to further increase the dose of T cells, may be needed in order to achieve better tumor control.

In the clinic, T cells are administered together with IL2 and after a lymphodepleting chemotherapy regimen, which have been shown to improve their in vivo engraftment potential [41, 42]. Since systemic exposure to high-dose IL2 often results in grade 3 or 4 adverse events in patients [43], we tested the combination of T cells with a tumor-targeted IL2, as a strategy to reduce exposure to healthy organs and focus IL2 activity at the site of disease. The strong anti-tumor activity of monotherapy with F8-IL2, together with the lack of synergistic effect when F8-IL2 was administered with T cells, indicated that the immunocytokine preferentially acted on T cells present in the tumor prior to adoptive cell transfer. Since in the clinic, preexisting TILs are depleted with chemotherapy, it would be of interest to investigate the effect of F8-IL2 on the persistence of adoptively transferred T cells after lymphodepletion. In addition, many other agents could potentially improve the engraftment, survival and anti-tumor activity of infused T cells. Our model may allow a systematic investigation of different combination strategies in a setting, which closely mimic the procedures already employed in the clinic.

Many preclinical studies have shown that minimally cultured, phenotypically younger T cells perform better in ACT, achieving higher anti-tumor effect at lower doses [28]. Considerable efforts in the field of ACT are being devoted to the development of new methods to control the phenotype of T cell products. Different strategies have been proposed to expand T cell with naïve- or T memory stem cell-like characteristics, including the use of anti-oxidants [44], the inhibition of Akt or p38 kinases [37, 45] or the enhancement of Wnt- or Notch-signaling [36, 38]. These experiments have so far been performed using naïve T cells (or in vitro activated T naïve, in one case) and may not be as effective on TILs. In our hands, expansion of TILs in the presence of GSK-3b or p38 inhibitors, as well as recombinant Notch-ligand, did not induce expression of CD62L, a marker of naïve and central-memory T cells. The AH1 model and our culture method could serve as a basis for the systematic investigation of new conditions for the reprogramming of antigen-experienced T cells to a younger phenotype.

AH1 has a number of characteristics, which make it a very attractive target for cancer therapy. It is highly expressed in the tumor but not in healthy organs, it is expressed in multiple tumors of different histological origin, and it is derived from a viral protein, which increases its chances to be immunogenic, as T cells are unlikely to have undergone negative selection against it in the thymus [46]. The presence of endogenous retroviral elements has been confirmed also in the human genome, and aberrantly expressed retroviral protein may represent a good source of tumor-specific antigen, analogously to AH1 [47]. Aberrantly expressed antigen as a whole has been shown to account for about 90% of the total tumor antigens in both mice and humans, and aberrantly expressed antigenic peptides other than AH1 have been identified in murine CT26 tumors [48, 49]. Some of these antigens may contribute to the rejection process. In fact, we could identify T cells specific for some of the identified CT26 antigens in tumors and TDLNs of tumor-bearing mice, which received L19-IL12 (Supp. Figure 10). Our protocol could be potentially used to expand and study those T cells, analogously to what has been done with AH1-specific T cells.

Protocols for the preparation of T cell therapeutics for ACT should aim at generating the highest number of phenotypically fit tumor-specific T cells. TILs have been originally chosen as a source of T cells for ACT, with the rational that a higher concentration of tumor-specific T cells may be found at the site of disease. It has been shown, however, that the fraction of T cells infiltrating human tumors and able to recognize malignant cells is variable and generally low [50]. Circulating T cells have recently been proposed as a promising alternative to TILs [5–8]. The proportion of tumor-specific T cells found in the blood is generally even lower than in the tumor; however, these T cells could be successfully expanded and were shown to accumulate in the tumor after re-infusion [5, 6, 16]. Tumor-specific T cells are enriched during the expansion protocol, but their content in the infusion bag is still variable [6, 13]. Since survival factors, nutrients and activating molecules are limited in vivo, the more tumor-specific T cells are infused, and the higher is their chance to engraft and exert their therapeutic potential. It may thus be desirable to perform ACT with pure populations of tumor-specific T cells. With our protocol, we were able to generate high numbers of pure AH1-specific T cells. Such protocol, together with methods to predict antigenic peptides, may be implemented for the expansion of tumor-specific T cells in the clinic.

### Supplementary Information

Below is the link to the electronic supplementary material.

Supplementary file1 (PDF 544 kb)
