# Supplementary material for: Cancer therapy in mice using a pure population of CD8+ T cell specific to the AH1 tumor rejection antigen
Source: Cancer Immunol Immunother. 2021 Apr 1;70(11):3183–97. doi: 10.1007/s00262-021-02912-9 (PMC8505334; doi:10.1007/s00262-021-02912-9)
Supplement: Supplementary file 1 — Supplementary file1 (PDF 544 kb) [file 262_2021_2912_MOESM1_ESM.pdf]

Supplementary Data

Supplementary Methods

Synthesis of MVP-compound and FITC-lated AH1-peptide

General Remarks

High-Resolution Mass Spectrometry (HRMS) spectra and analytical Reversed-Phase Ultra Performance Liquid Chromatography (UPLC) were recorded on a Waters Xevo G2-XS QTOF coupled to a Waters Acquity UPLC H-Class System with PDA UV detector, using an ACQUITY UPLC BEH C18 Column, 130 Å, 1.7 µm, 2.1 mm × 50 mm at a flow rate of 0.6 ml/min with linear gradients of solvents A and B (A = Millipore water with 0.1% FA, B = MeCN with 0.1% FA). Preparative reversed-phase high-pressure liquid chromatography (RP-HPLC) were performed on a Waters Alliance HT RP-HPLC with PDA UV detector, using a Synergi 4µm, Polar-RP 80Å 10 × 150 mm C18 column at a flow rate of 4 ml/min with linear gradients of solvents A and B (A = Millipore water with 0.1% TFA, B = MeCN with 0.1% TFA).

All compounds and chemical reagents were obtained from Sigma-Aldrich and TCI Europe and used without further purification. The AH1- and the biotinylated-peptide scaffold were purchased from Biomatik.

Synthesis of MVP-compound

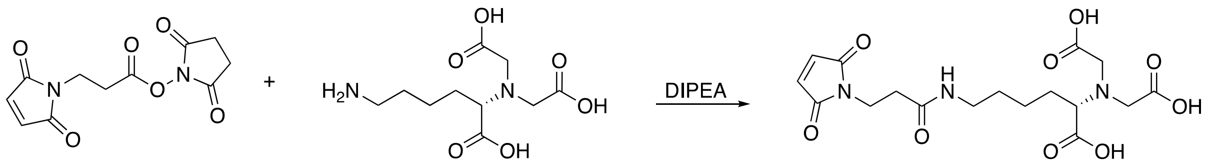

Supp. Figure 1. Functionalization of NTA-Lysine with 3-(Maleimido)propionic acid N-hydroxysuccinimide ester.

NTA-Lysine (60 mg, 0.229mmol, 1eq) was dissolved in 1 mL of dry-DMSO in presence of DIPEA (d=0.742, 160uL, 4eq). In another vial, 3-(Maleimido)propionic acid N-hydroxysuccinimide ester (54 mg, 0.9 eq) was dissolved in dry-DMSO. The solution was added to the vial and let react at 35°C, until no starting-material was detected on UPLC-MS (ca. 1 hour). The mixture was purified directly on reverse-phase HPLC (Synergi RP Polar,

5% MeCN in 0.1% aq. TFA to 70% over 14 min), to obtain 25 mg of a white pure compound (30% yield).

HRMS (ES) calculated for $[M+H]^+$ (m/z): 414.1507, found 414.1581.

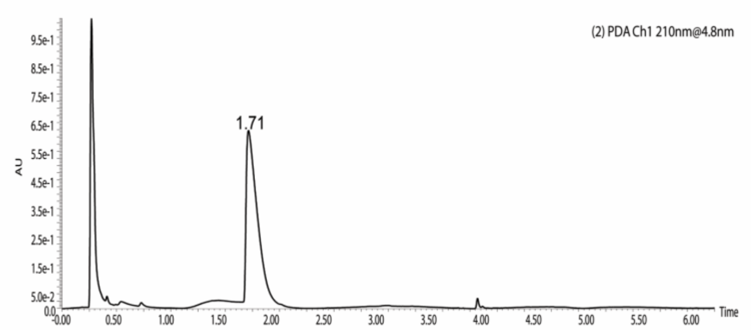

Supp. Figure 2. UPLC-profile of the purified product.

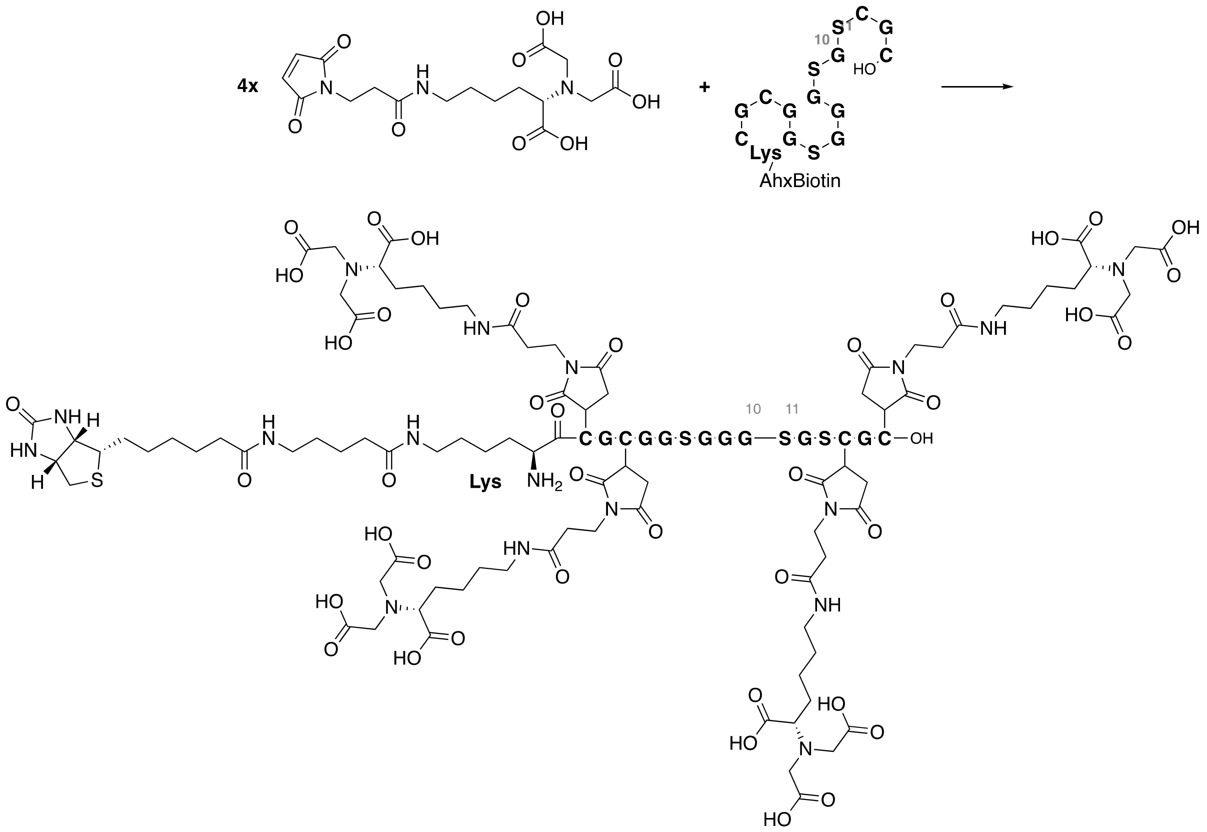

Supp. Figure 3. Synthesis of MVP compound.

Biotinylated peptide scaffold (10 mg, 1 eq) with the following sequence:

(Biotin-NεLys)-CGCGGSGGGSGSCHC-COOH

was dissolved in DMSO at 0.1M concentration in presence of MVP_1 compound (8 eq, 0.1 M in DMSO). The reaction was kept at 37°C for 5 hours until no starting material was detected by UPLC-MS analysis. The mixture was purified on reverse-phase HPLC (Synergi

RP Polar, 5% MeCN in 0.1% aq. TFA to 50% over 14 min), to obtain 5 mg of a white compound (25% yield).

HRMS (ES) calculated for $[M+H]^{2+}$ (m/z): 1635.0817, found 1635.5149.

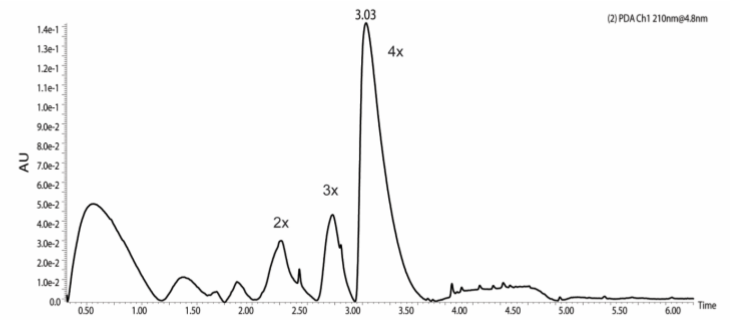

Supp. Figure 4. UPLC-profile of the purified MVP-compound (low UV-absorption).

Synthesis AH1-FITC

AH1 peptide (10 mg, 1 eq) was dissolved in 100 μL of dry-DMSO in presence of DIPEA (1 eq). Fluorescein isothiocyanate (1 eq, FITC) was added portion-wise at room temperature from a 0.1 M solution in dry-DMSO until neither starting material nor free-FITC were detected by UPLC-MS analysis. The mixture was purified on reverse-phase HPLC (Synergi RP Polar, 5% MeCN in 0.1% aq. TFA to 80% over 14 min), to obtain 6 mg of a yellow powder (45% yield).

HRMS (ES) calculated for $[M+H]^{2+}$ (m/z): 1517.5582, found 1517.4576

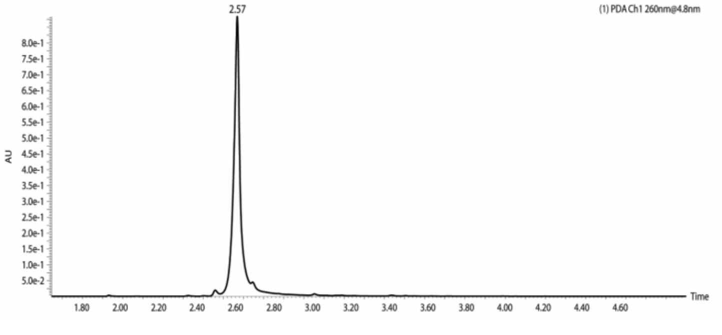

Supp. Figure 5. UPLC-profile of the purified FITC-lated AH1-peptide.

Supplementary Results

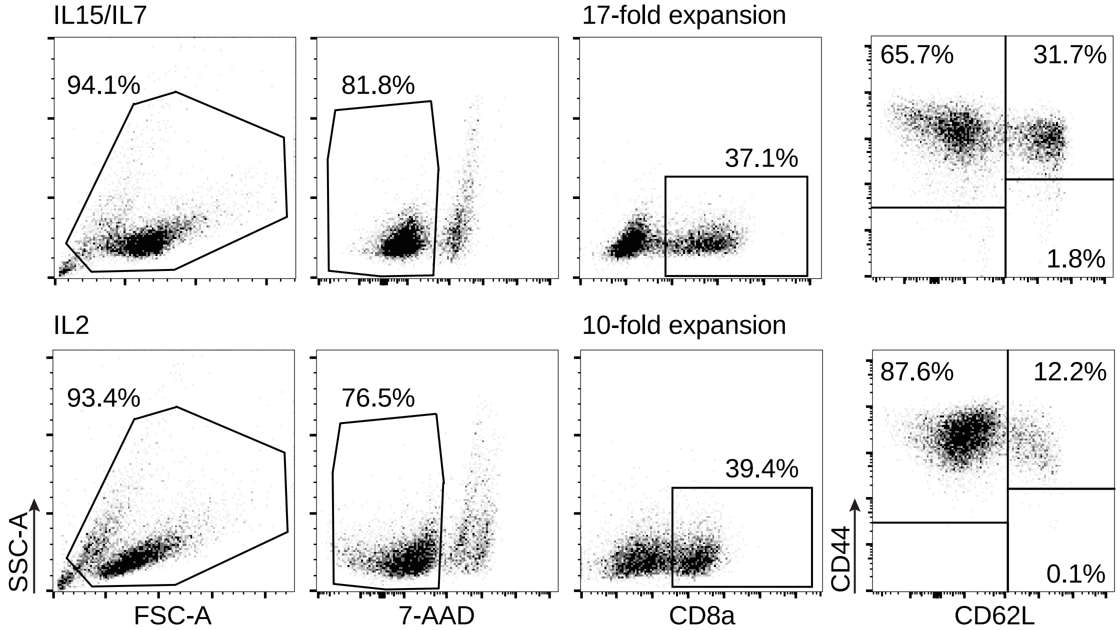

Supplementary Figure 6. Culture of CD90.2+ splenocytes in IL2 vs IL15/IL7. Flow cytometry analysis of CD90.2+ splenocytes, purified using MojoSort Mouse CD90.2 Selection Kit (Biolegend) and cultured in CM containing Dynabeads Mouse T-activator CD3/CD28 (Thermofisher, 1:1 Dynabeads to T cells) and either 60 IU/mL human IL2 (Proleukin, Novartis), or 10ng/mL of both recombinant murine IL15 and IL7 (Biolegend) for 14 days. Half of the medium containing corresponding cytokines was replaced every 3-4 days. For culture in IL2 only, new Dynabeads were added every 5-6 days.

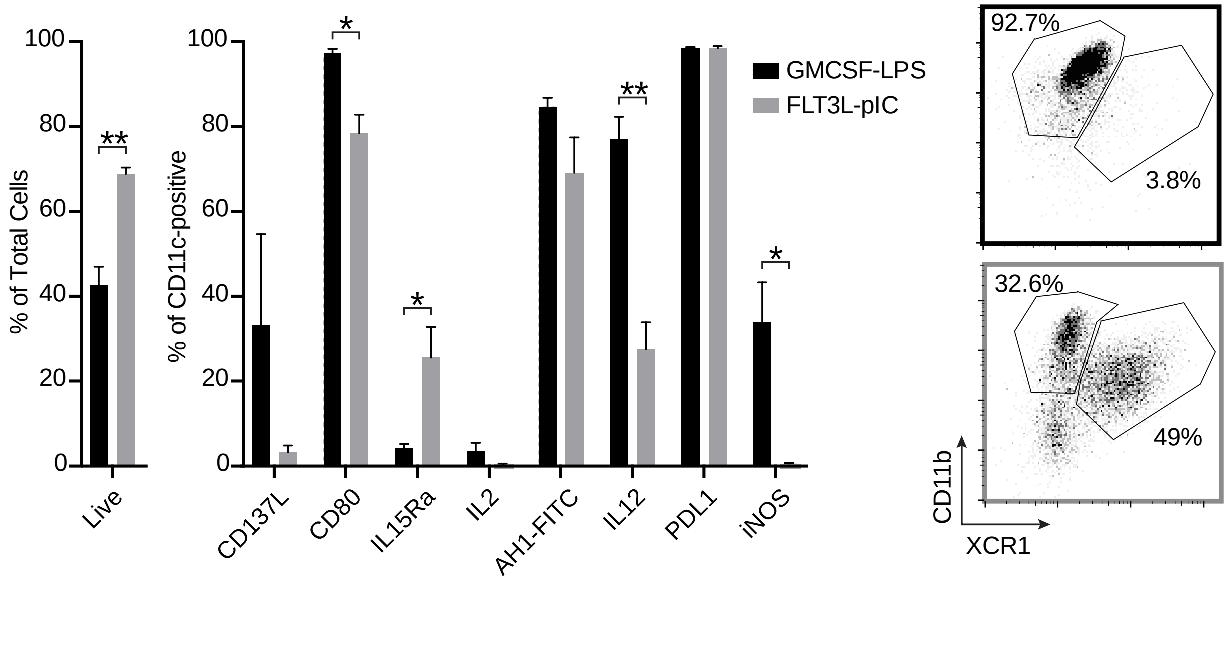

Supplementary Figure 7. Characterization of Bone marrow-derived Dendritic Cells (BMDCs).

BMDCs cultured either in GM-CSF or Flt3L and matured with LPS resp. poly I:C, were pulsed with AH1 peptide (or a FITC-lated version of the AH1-peptide) and expression of various markers was characterized by flow cytometry. Columns represent means ± SEM, n = 3 biological replicates for each experimental condition, * = $p < 0.05$, ** = $p < 0.01$, *** p = < 0.001, **** = $p < 0.0001$ (unpaired t-test).

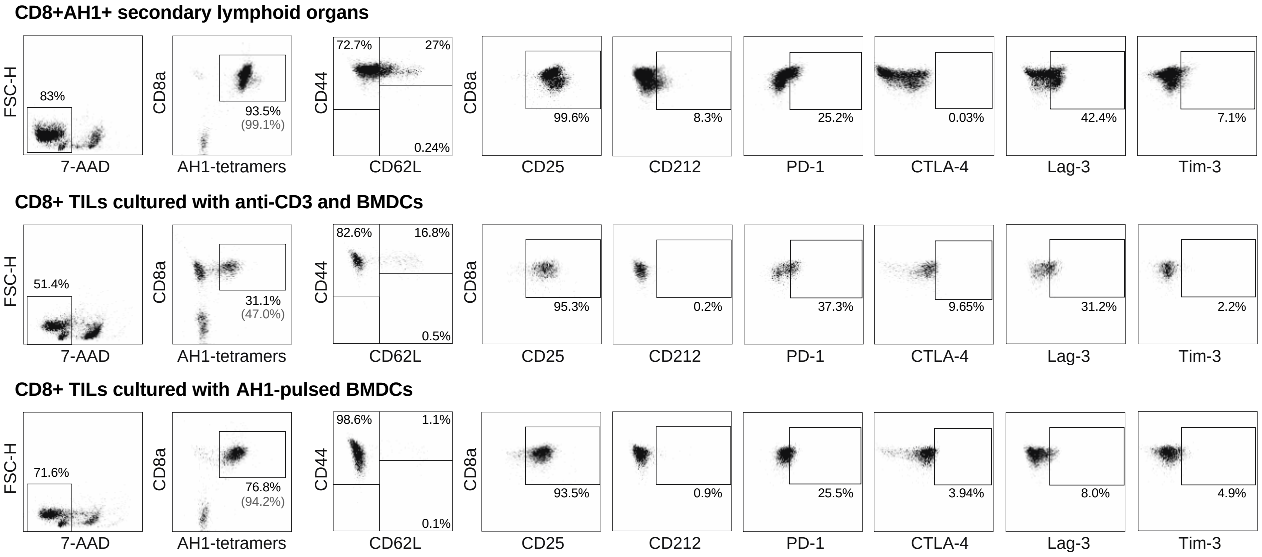

Supplementary Figure 8. Characterization of AH1-specific T cells from secondary lymphoid organs, resp. CD8+ TILs.

FACS-purified, AH1-multimers specific CD8+ T cells from secondary lymphoid organs, or bulk CD8+ TILs were expanded using our optimized protocol, or a slightly modified protocol employing non-pulsed BMDCs and anti-CD3 antibody (145-2C11, Biolegend). Sample were analysed by flow cytometry after 3 weeks of *in vitro* culture. Percentages of living cells, of AH1-tetrmers positive CD8+ T cells over living cells (black) and over living CD8+ T cells (grey, in brackets), phenotype of CD8+ T cells, as well as percentages of CD8+ T cells, which stained positive for selected activation or exhaustion markers, are shown.

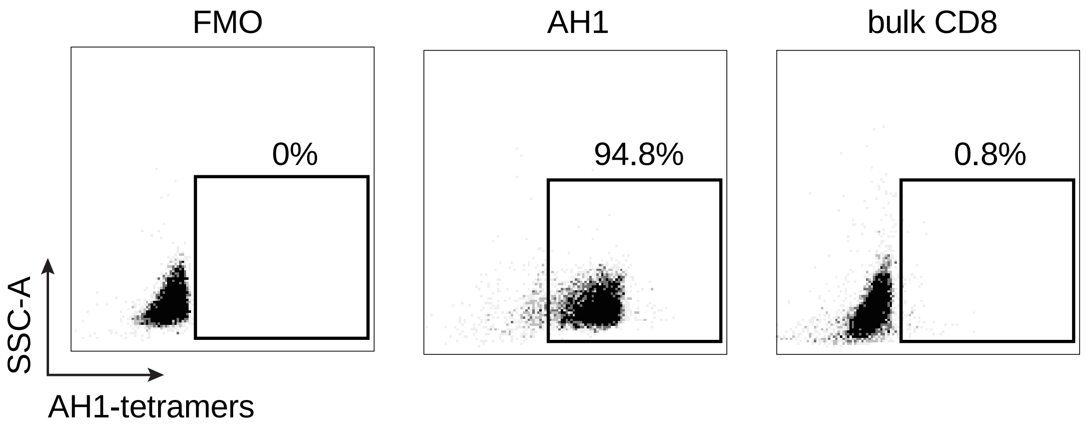

Supplementary Figure 9. AH1-tetramers specific CD8+ T cells in "bulk CD8".
AH1-multimers negative CD8+ T cells from secondary lymphoid organs (bulk CD8) were purified by FACS and cultured with the modified protocol employing non-pulsed BMDCs and anti-CD3 antibody instead of AH1-pulsed BMDCs. After 3 weeks of *in vitro* culture, T cells were analysed for reactivity against AH1-tetramers. AH1+CD8+ T cells isolated the same way, but expanded with the normal protocol (AH1), were used as positive control.

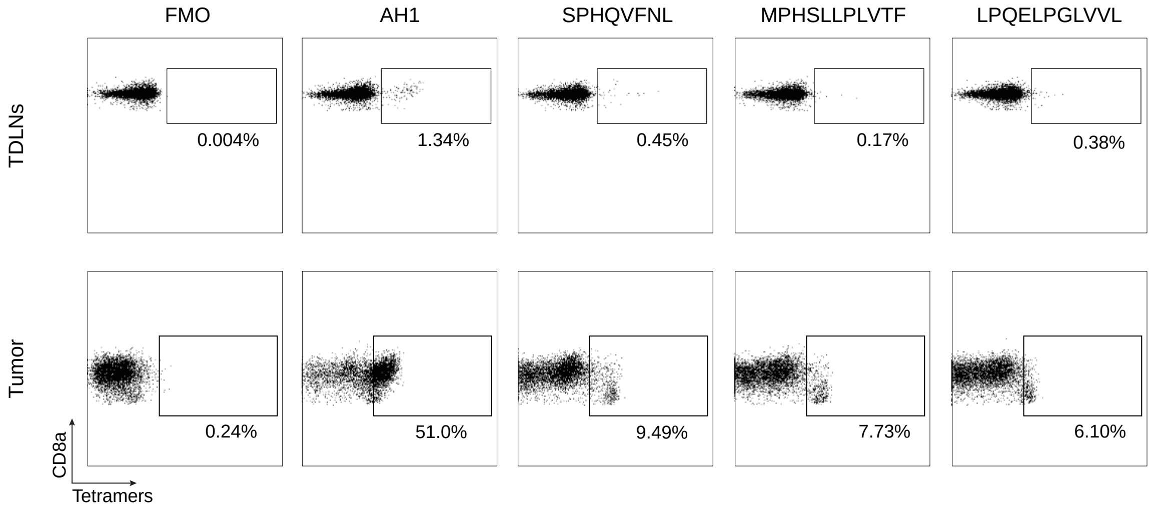

Supplementary Figure 10. T cells specific for additional CT26-derived antigenic peptides
Flow cytometry analysis of CD8+ T cells from tumor and tumor-draining lymph nodes (TDLNs). Cells were stained with H-2L^d-tetramers loaded with the AH1 peptide (AH1) or with other three CT26-derived peptides (amino acids sequence provided) identified in a previous study[1].

1. Laumont, C.M. et al. Noncoding regions are the main source of targetable tumor-specific antigens. *Sci Transl Med* **10** (2018).
